# HPV integration: a precise biomarker for detection of residual/recurrent disease after treatment of CIN2-3

**DOI:** 10.1186/s13027-024-00600-8

**Published:** 2024-08-08

**Authors:** Fanwei Huang, Liang He, Wei Li, Xiaoyuan Huang, Tao Zhang, Munawaer Muaibati, Hu Zhou, Shimin Chen, Wenhui Yang, Fan Yang, Liang Zhuang, Ting Hu

**Affiliations:** 1 National Clinical Research Centre for Obstetrics and Gynaecology, Cancer Biology Research Centre (Key Laboratory of the Ministry of Education), Tongji Hospital, Tongji Medical College, Huazhong University of Science and Technology, Wuhan, China; 2 Department of Gynecology and Obstetrics, Tongji Hospital, Tongji Medical College, Huazhong University of Science and Technology, Wuhan, China; 3 New Technology Platform, Wuhan KDWS Biological Technology Co., Ltd., Wuhan, China; 4 Cancer Center, Tongji Hospital, Tongji Medical College, Huazhong University of Science and Technology, Wuhan, China

**Keywords:** Human papillomavirus, Cervical intraepithelial neoplasia, Recurrence, Human papillomavirus integration, Follow-up

## Abstract

**Background:**

This study aimed to investigate whether persistent human papillomavirus integration at the same loci (PHISL) before and after treatment can predict recurrent/residual disease in women with CIN2-3.

**Methods:**

A total of 151 CIN2-3 women treated with conization between August 2020 and September 2021 were included. To investigate the precision of HPV integration, we further analyzed HPV integration-positive patients. Sensitivity, specificity, positive and negative predictive values (PPV and NPV, respectively), and the Youden index for predicting recurrence/residual disease were calculated.

**Results:**

Among the 151 enrolled CIN2-3 women, 56 were HPV integration-positive and 95 had HPV integration-negative results. Six (10.7%) experienced recurrence among 56 HPV integration-positive patients, which was more than those in HPV integration-negative patients (one patient, 1.1%). In the 56 HPV integration-positive patients, 12 had positive HPV results after treatment, seven had PHISL, and two had positive cone margin. Among the seven patients who tested with PHISL, six (85.7%) had residual/recurrent disease. PHISL was a prominent predictor of persistent/recurrent disease. The HPV test, the HPV integration test, and PHISL all had a sensitivity of 100% and a NPV of 100% for residual/recurrent disease. PHISL showed better specificity (98.0% vs. 82.0%, *p* = 0.005) and PPV (85.7% vs. 40.0%, *p* = 0.001) than the HPV test for predicting recurrence.

**Conclusions:**

The HPV-integration-positive CIN2-3 women had much higher relapse rates than HPV-integration-negative CIN2-3 women. The findings indicate that PHISL derived from preoperative and postoperative HPV integration tests may be a precise biomarker for the identification of residual/recurrent CIN 2/3.

**Supplementary Information:**

The online version contains supplementary material available at 10.1186/s13027-024-00600-8.

## Background

The development of CIN2-3 or worse is highly correlated with persistent high-risk human papillomavirus (HPV) infection [1]. Effective treatment not only involves removing the lesion but also treating the underlying HPV infection [2–4]. Despite treatment, about 15% (range 5–25%) of patients are diagnosed with cervical high-grade disease after conization due to residual or recurrent lesions [2, 5–8]. Loss of follow-up is a significant factors in patients developing cervical cancer after receiving prior CIN treatment [9].

Due to this substantial risk, patients are kept under close surveillance after treatment. The 2019 American Society for Colposcopy and Cervical Pathology (ASCCP) guidelines for the management of patients treated for CIN2-3 suggested HPV-based testing at 6 months after conization. If an HPV-based test is positive, colposcopy and biopsy are recommended [10]. Although HPV infection is the primary contributor to cervical cancer, 80%–90% of HPV infections are transient, with only 10–20% of individuals developing a persistent infection [11]. According to previous research, HPV testing has a sensitivity and NPV of nearly 98% for predicting recurrence but a relatively low specificity (about 80%) and positive predictive value (PPV) (20–60%) [8, 12, 13]. Both the 2019 ASCCP guidelines [10] and the Chinese Expert Consensus on Cervical Cancer Screening and Abnormal Management [14] recommend HR-HPV testing at 6 months postoperatively and suggest colposcopy for women with positive HPV results. Moreover, many patients have a long-term natural regression time after CIN2-3 treatment [15]. Accordingly, an HPV test result-driven screening program would inevitably lead to high false-positive rates, which would result in unnecessary colposcopies and cervical biopsies.

As such, colposcopy makes for considerable psychological stress and physical discomfort and may pose a potential risk of overtreatment, increasing the psychological and financial stress of patients [10]. This is quite inefficient since only 75–95% of the patients with positive HPV results after surgery will not have recurrent/residual disease [16, 17]. Therefore, a test that can accurately predict recurrent/residual disease after conization is urgently needed.

Given the continuous advancement of high-throughput sequencing technology, research has revealed that cervical cancer development is a long-term process. In this respect, the integration of the HPV DNA into the host genome has been reported to be a crucial step in cervical carcinogenesis, as HPV DNA integration continues to express oncoproteins and increase the instability of the host genome, inducing the progression of the normal cervical epithelium into precancerous lesions and eventually cancer [18–20]. It is now recognized that HPV genomic integration is an important marker for the malignant transformation of cervical intraepithelial neoplasia into cervical cancer [18, 21].

In benign lesions caused by HPV, intracellular viral DNA is usually located outside the host genome, whereas in high-grade intraepithelial neoplasia and cervical cancer, HPV DNA is typically integrated into the host genome, and the integration rate increases significantly with the severity of cervical lesions [22–24]. For instance, a previous high-throughput sequencing study found that although HPV integration events occurred in both precancerous lesions and cervical cancer samples, the statistical findings demonstrated that the incidence of HPV integration events was significantly higher in cervical cancer samples (approximately 95%) than in CINs samples (high level of a discrepancy) [25–27]. This suggests that HPV integration events occurred in an incremental process from precancerous lesions to cancerous lesions.

Various studies have demonstrated that persistent HR-HPV infection and integration of viral DNA into the human genome are the key events in cervical carcinogenesis [21]. In our preliminary experiments, a new technique that allows for the precise localization of integration loci information (human and virus-specific integration sites) was developed by conducting genome-wide HPV integration mapping in cervical cancer, which provided powerful technical support for our research [28]. Since the occurrence of HPV integration into the human genome is random, each individual’s integration site is unique [18]. Theoretically, persistent human papillomavirus integration at the same loci (PHISL) allows for the accurate prediction of the presence of post-surgical residuals. In this study, we investigated whether PHISL could be used as a biomarker predictive for recurrence after CIN2-3 treatment in order to identify a new screening protocol with higher accuracy and minimal impairment to patients.

This study’s objective was to evaluate PHISL’s potential as a brand-new predictor of CIN2-3 persistence or recurrence after conization. Additionally, we intended to contrast it with the other predictors of residual or recurrent disease following CIN2-3 therapy.

## Methods

### Study design

**Fig. 1:**
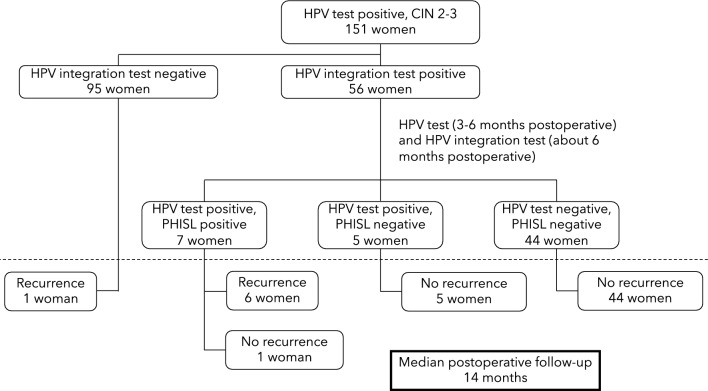
The flowchart of study enrollment. HPV, human papillomavirus; CIN, cervical intraepithelial neoplasial; LEEP, loop electrosurgical procedure; CKC, cold knife conization. ^#^ Figure can be placed near the section “Study design”. Figure was created using Microsoft PowerPoint

This is an observational cohort study that included 151 consecutive women diagnosed with CIN2-3 lesions who underwent conization by loop electrosurgical excision procedure (LEEP) or cold knife conization (CKC) in the Department of Obstetrics and Gynecology of Tongji Hospital in Wuhan between August 2020 and September 2021. A flowchart of the study protocol is depicted in Fig. 1. All patients underwent an HPV test 3–6 months postoperatively. Patients who had positive HPV results after treatment were advised to receive colposcopies and biopsies (if necessary), and were kept under close surveillance. Additionally, all HPV-positive cases were prospectively tested for HPV integration (by conducting whole-genome sequencing and high-throughput viral integration detection) at approximately 6 months after conization. Women with other histological diagnoses, women with a history of conization, women who underwent re-excision or hysterectomy shortly following LEEP/CKC, women lacking follow-up information, and immunosuppressed women were all excluded from the study. Written informed consent was obtained from each participant in this cohort study, which was approved by the Ethics Committee of Tongji Hospital of Huazhong University of Science and Technology (TJ-IRB20211110).

### HPV testing

Cervical cellular material was collected using a cytology brush and placed in a vial with a preservation solution for the HPV DNA test (Cobas® 4800 Test, Roche Molecular Systems, Alameda, CA). According to the manufacturer’s recommendations, Cobas 4800 technology was applied to process each sample. Fourteen HR-HPV genotypes were detected by Cobas 4800, reported as HPV16, HPV18 and “other HR-HPVs” (HPV31/33/35/39/45/51/52/56/58/59/66/68).

### HPV integration detection

The HPV integration test was performed using high-throughput viral integration detection (HIVID) according to our previous study [28]. For potential HPV integration sites identified by HIVID, the region of interest was verified by PCR and Sanger sequencing. This next-generation sequencing-based test also detected HPV infection types. Samples with more than five supporting reads were considered HPV integration-positive. Samples with discrepant HPV infection results between the HPV integration test and Cobas were eliminated.

### Surgical procedure

The types of conization included were LEEP and CKC, and those cases selected to undergo the procedure were evaluated by the surgeon. During LEEP conization, the cervix was exposed using an adapted speculum for smoke evacuation and a high-frequency electrical generator was used for cutting and coagulation. Local anesthesia was administered by injecting 2% lidocaine plus epinephrine in each quadrant of the cervix. The size of the loop was selected according to the size of the area to be excised. Under general anesthesia, CKC was performed in the operating room to completely remove the visible lesion. The cone’s vertical dimensions varied from 1.5 to 2.5 cm.

All specimens were anatomically oriented and fixed in 10% formalin medium subsequently. If the lesion was within 1 mm of the resection surface or reached it, the margin was deemed positive.

### Follow-up

Post-treatment follow-up visits took place at 6-month intervals during the initial 2 years following conization and annually thereafter, with an HPV test and cervical cytology performed at each visit. The initial visit was set for 4–6 months after surgery if the patient was determined to have positive surgical margins. Patients with abnormal cytology (CIN1+) or abnormal colposcopy underwent a colposcopically directed biopsy. When the transformation zone was partially obscured or no abnormality was detected, an endocervical curettage (ECC) was performed.

### Criteria for the diagnosis and treatment of residual/recurrent disease

Residual/recurrent disease was defined as CIN2-3 or worse diagnosed by histological confirmation. The diagnosis of recurrent or residual CIN1 was confirmed according to the results of colposcopically directed biopsy or ECC. Patients with the histological confirmation of CIN2+ during follow-up underwent a second surgery, while patients with histologically confirmed CIN1 were followed up by the study protocol without therapy, except for those who had endocervical involvement.

### Statistical analysis

Data are summarized using proportions and medians (ranges). The correlation between residual or recurrent disease with cone margin status and laboratory tests (HPV tests and HPV integration detection) was determined using the Chi-squared test or Fisher’s exact test. By calculating the sensitivity, specificity, PPV, NPV, and Youden index for predicting residual/recurrent CIN2-3, we evaluated the diagnostic efficacy of these tests. Differences in positivity, sensitivity, and specificity were evaluated using an exact McNemar’s Chi-squared, and differences in PPV and NPV were evaluated using the method developed by Leisenring and Pepe using the “DTComPair” package in R. All the stated p-values were two-sided and regarded as statistically significant at *p* < 0.05. SPSS for Windows version 18.0 (SPSS Inc., Chicago, IL, USA) and R (version 3.3.1) were used for statistical analysis.

## Results

### Patient characteristics

The patient characteristics are listed in Table 1. The 151 patients included in the study had a median age of 37 years (range 21–66 years), and 18 (11.9%) patients were postmenopausal. Histological analysis of the 151 biopsy specimens confirmed CIN-2 in 51 (33.8%) and CIN-3 in 100 (66.2%) patients. There were 3 patients (2.0% of the total) with positive cone margin status, 2 of 56 (3.6%) with HPV integration-positive results, and 1 of 95 (1.1%) with HPV integration-negative results. Pre-treatment HPV tests by Cobas 4800 were positive in all 151 patients, of which HPV-16 (47.7%) was the most prevalent HR-HPV type. HPV integration test before conization showed similar results, with HPV-16 being the most common integrated type followed by HPV 52, 58, 33, 18, 31, 53, 35, 39, 56, 59, 66, 82, 51, 68, 45, 26, and 73.

**Table 1:** Patient characteristics (n = 151)

Characteristics	Parameters	Values		
		HPV integration positive	HPV integration negative	Total
Age (years)	Median (Range)	41.5 (24–66)	35 (21–60)	37 (21–66)
Parity (times)	Median (Range)	1 (0–3)	1 (0–3)	1 (0–3)
Menopause	No	39 (69.6%)	91 (95.8%)	130 (86.1%)
	Yes	14 (25.0%)	4 (4.2%)	18 (11.9%)
	Not available	3 (5.4%)	0	3 (2.0%)
HPV test (preoperative, Cobas 4800)	HPV16 +	35 (62.5%)	37 (38.9%)	72 (47.7%)
	HPV18 +	5 (8.9%)	5 (5.3%)	10 (6.6%)
	Other types of HPV +	26 (46.2%)	67 (70.5%)	93 (61.6%)
HPV types (preoperative, HPV integration detection)	16	34 (60.7%)	38 (40.0%)	72 (47.7%)
	52	15 (26.8%)	22 (23.2%)	37 (24.5%)
	58	4 (7.1%)	26 (27.4%)	30 (19.9%)
	33	3 (5.4%)	13 (13.7%)	16 (10.6%)
	18	5 (8.9%)	4 (4.2%)	9 (6.0%)
	31	2 (3.6%)	5 (5.3%)	7 (4.6%)
	53	2 (3.6%)	3 (3.2%)	5 (3.3%)
HPV types (preoperative, HPV integration detection)	35	2 (3.6%)	2 (2.1%)	4 (2.6%)
	39	2 (3.6%)	1 (1.1%)	3 (2.0%)
	56	1 (1.8%)	2 (2.1%)	3 (2.0%)
	59	1 (1.8%)	2 (2.1%)	3 (2.0%)
	66	1 (1.8%)	2 (2.1%)	3 (2.0%)
	82	1 (1.8%)	1 (1.1%)	2 (1.3%)
	51	1 (1.8%)	1 (1.1%)	2 (1.3%)
	68	0	2 (2.1%)	2 (1.3%)
	45	0	2 (2.1%)	2 (1.3%)
	26	1 (1.8%)	0	1 (0.7%)
	73	0	1 (1.1%)	1 (0.7%)
Pathology	CIN2	11 (19.6%)	40 (42.1%)	51 (33.8%)
	CIN3	45 (80.4%)	55 (57.9%)	100 (66.2%)
Cone margin status	Negative	54 (96.4%)	94 (98.9%)	148 (98.0%)
	Positive	2 (3.6%)	1 (1.1%)	3 (2.0%)
Residual/recurrent cases	CIN2	1 (1.8%)	0	1 (0.7%)
	CIN3	5 (8.9%)	1 (1.1%)	6 (4.0%)

HPV, human papillomavirus; CIN, cervical intraepithelial neoplasia

Values are presented as median (range) or number (%)

#Table can be placed near the section “Patient Characteristics”

Of the 151 patients, 7 (4.6%) had residual/recurrent disease during the follow-up. There were six patients with CIN-3 (4.0%) and one patient with CIN-2 (0.7%) residual/recurrent lesions.

The median postoperative follow-up for all patients was 12 months (range 6–27 months). The characteristics of patients who had recurrences during the follow-up during the follow-up with a median time of 5.5 months (range 3–7 months) are shown in Table 2. Of the remaining eight HPV-positive patients who did not relapse, the HPV infection of five patients spontaneously cleared within a median time of 9 months, and the other three maintained positive HPV results throughout the follow-up period, with a median follow-up time of 12 months. None of the patients who tested negative for HPV at the first postoperative follow-up developed residual or recurrent disease.

**Table 2:** Characteristics of women who tested HPV-positive at the first follow-up (3–6 months)

Cases	Age (years)	Initial treatment modality	Recurrence		HPV test			HPV integration test^*^	
			Yes/No	Time from treatment to recurrence (months)	3–6 months after treatment	Time from treatment to HPV clearance (months)	Latest results after treatment	Before treatment	After treatment
1	44	LEEP	Yes	7	HPV16 +	–	–	HPV16 +	HPV16 +
2	63	CKC	No	–	HPV16 +	–	HPV16 +	HPV16 +	HPV16 +
3	37	LEEP	Yes	5	HPV16 +	–	–	HPV16 +	HPV16 +
4	52	LEEP	Yes	3	Other 12 types	–	–	HPV33 + , HPV52 +	HPV33 +
5	59	LEEP	No	–	Other 12 types	–	Other 12 types	HPV52 +	HPV52 +
6	37	LEEP	No	–	HPV16 +	–	HPV16 +	HPV16 +	HPV16 +
7	54	LEEP	Yes	3	HPV16 +	–	–	HPV16 +	HPV16 +
8	35	LEEP	Yes	6	HPV16 +	–	–	HPV16 +	HPV16 +
9	30	LEEP	No	–	Other 12 types	16	Negative	HPV16 + , HPV52 + , HPV58 +	Negative
10	47	CKC	No	–	HPV16 +	11	Negative	HPV16 +	Negative
11	46	LEEP	No	–	HPV16 +	9	Negative	HPV16 +	Negative
12	61	LEEP	Yes	6	Other 12 types	–	–	HPV51 +	HPV51 +

HPV, human papillomavirus; LEEP, loop electrosurgical procedure; CKC, cold knife conization

*Type of HPV genome integration

#Table can be placed near the section “The correlation between residual/recurrent disease and postoperative HPV tests, HPV integration tests and cone margin status in HPV integration-positive patients”

### Correlation between preoperative HPV integration status and residual/recurrent diseases

Among the 151 enrolled CIN 2–3 women, 56 women were HPV integration-positive and 95 women had HPV integration-negative results. Six (10.7%) women experienced recurrence in 56 HPV integration-positive patients, which were more than those in HPV integration-negative patients (one patient, 1.1%).

Considering that 85.7% (6/7) of residue lesions were HPV-integration-positive before surgery and these lesions were all HPV-integration-positive at the same loci after surgery, we conducted further investigations in HPV-integration-positive women in the following analysis.

### Correlation between residual/recurrent disease and postoperative HPV tests, HPV integration tests, and cone margin status in HPV integration-positive patients

At the first follow-up after conization, 12 out of the 56 HPV integration-positive patients (21.4%) tested positive for HPV (Table 2). Among the 12 patients, nine were positive for HPV integration, of whom seven were PHISL and the other two were non-PHISL. Among the seven patients with PHISL, six (85.7%) patients were infected with a single HR-HPV type, and HPV-16 (71.4%) was the most prevalent HR-HPV infection type (Table S1). The cone margins were involved in two women (3.6%), one of whom had recurrence.

Interestingly, PHISL was detected in all six patients who developed recurrence, with a median time of 6 months between the two integration tests, indicating that persistent HPV integration may be an independent biomarker that accurately predicts residual/recurrent disease following conization.

Postoperative HPV status and PHISL both had a significantly positive association with residual/recurrent CIN2-3 (*p* < 0.001, Table 3). A significantly higher risk of residual/recurrent CIN2-3 was linked to HPV-16 or HPV-18 infection (*p* = 0.003). Age and margin status were not associated with residual/recurrent CIN2-3 (Table 3).

**Table 3:** Factors predicting recurrence after treatment in CIN2-3 patients

Factor		Recurrence (n = 6)	No recurrence (n = 50)	*P* value^*^
Cone margin status	Negative	5 (83.3)	49 (98.0)	n.s
	Positive	1 (16.7)	1 (2.0)	
HPV test	Negative	0 (0.0)	44 (88.0)	< 0.001
	Positive	6 (100.0)	6 (12.0)	
HPV16 or HPV18 infection	Negative	2 (33.3)	46 (92.0)	0.003
	Positive	4 (66.7)	4 (8.0)	
HPV integration	Negative/Persistent HPV integration at different breakpoint	0 (0.0)	49 (98.0)	< 0.001
	Persistent HPV integration at the same breakpoint	6 (100.0)	1 (2.0)	
Single or multiple infection	Single infection	5 (83.3)	34 (68.0)	n.s
	Multiple infection	1 (16.7)	16 32.0)	

HPV, human papillomavirus; n.s., not significant

Results are absolute numbers (%)

*P values from Fisher’s exact test

#Table can be placed near the section “The correlation between residual/recurrent disease and postoperative HPV tests, HPV integration tests and cone margin status in HPV integration-positive patients”

### The accuracy of HPV status, HPV16/18 genotyping, HPV integration, PHISL, multiple infections of HR-HPV, and cone margin for the detection of residual/recurrent disease

Table 4 shows the sensitivity, specificity, PPV, NPV, and the Youden index of HPV status, HPV16/18 genotyping, HPV integration status, PHISL, multiple infections of HR-HPV, and cone margin results for residual/recurrent disease after conization. For the detection of residual/recurrent disease, HPV tests, HPV integration, and PHISL all achieved 100% sensitivity and a negative predictive value. PHISL had a higher specificity (98.0%, *p* = 0.005), higher PPV (85.7%, *p* = 0.001), and higher Youden index (0.98) compared with HPV tests (82.0%, 40.0%, 0.82, respectively) and HPV integration (92.0%, 60.0%, 0.92, respectively).

**Table 4:** Performance of the six predictors for recurrence of HSIL

Factors	Sensitivity			Specificity			PPV			NPV			Youden Index
	%	95% CI	*P* value	%	95% CI	*P* value	%	95% CI	*P* value	%	95% CI	*P* value	
HPV test	100	–	Reference	82.0	71.4–92.6	Reference	40.0	15.2–64.8	Reference	100	–	Reference	0.82
HPV integration at the same site	100	–	–	98.0	94.1–100	0.005	85.7	59.8–100	0.001	100	–	< 0.001	0.98
HPV integration	100	–	–	92.0	84.5–99.5	0.025	60.0	29.6–90.4	0.025	100	–	< 0.001	0.92
Cone margin status	16.7	0–46.5	0.025	98.0	94.1–100	0.011	50.0	0–100	0.774	90.7	83.0–98.5	0.019	0.15
HPV16 or HPV18 infection	66.7	28.9–100	0.157	30.0	17.3–42.7	< 0.001	10.3	0.7–19.8	0.006	88.2	72.9–100	0.14	− 0.03
Multiple types of HR-HPV	16.7	0–46.5	0.025	68.0	55.0–80.9	0.108	5.9	0–17.1	0.009	87.2	76.7–97.7	0.018	− 0.15

PPV, positive predictive value; NPV, negative predictive values; CI, confidence interval; HPV, human papillomavirus; HR-HPV, high-risk human papillomavirus

#Table can be placed near the section “The accuracy of HPV status, HPV16/18 genotyping, HPV integration, PHISL, multiple infections of HR-HPV and cone margin for detection of residual/recurrent disease”

## Discussion

At present, HPV screening for predicting recurrence is the most commonly used method since the majority of viruses are cleared by the immune system within 2 years after conization [29]. HPV testing at the first follow-up (6 months after conization) in our study possessed high sensitivity and NPV (both 100%), and a specificity of 82.0%, but a relatively low PPV of 40%, respectively, which is similar to that reported by previous studies [30, 31]. HPV genotypes have also been employed as a tool to detect post-treatment recurrence [13]. The proportion of HPV16 was significantly higher than other genotypes both in the preoperative HPV integration test (60.7%) and in PHISL (71.4%). We also observed an association between CIN2-3 recurrence and HPV16/18 infection.

Previous studies have shown that approximately two-thirds of non-recurrent CIN2-3 patients tested negative for HPV within 1 year after successful conservative treatment, and in the remaining one-third of patients viral clearance continued at diminishing rates and was virtually complete at the end of the third year. In present study, of the 12 patients who tested positive for HPV at the first postoperative test, approximately half of them turned negative at 9–18 months after conization, most likely due to spontaneous regression. For these patients, such as the one in the case report, who remained HPV-positive 6 months after conization and cleared spontaneously within 1 year postoperatively, the current follow-up schedule in the clinic might result in unnecessary colposcopy examinations and treatments, whereas HPV integration testing may be more efficient and harmless.

HR-HPV testing at 6 months postoperatively and colposcopy for women with a positive HPV result are both recommended by the 2019 ASCCP guidelines [10] and the Chinese Expert Consensus on Cervical Cancer Screening and Abnormal Management [14]. Approximately 75–95% of patients with positive HPV results after surgery do not develop recurrent/residual disease [16, 17], which would necessitate unnecessary colposcopy and cervical biopsy. We identified PHISL as a biomarker for recurrence prediction following CIN2-3 therapy, and this novel screening method is more precise and less harmful to patients.

Our results indicate that PHISL is an efficient predictor for the recurrence of CIN2-3, suggesting that this approach is feasible and emphasizing the need for s further research to confirm these promising findings. The small sample size is one of our study’s primary weaknesses. The short follow-up time for some patients described in our study represent another drawback. Given that most relapses occur in the first 24 months, it is essential to validate these discoveries in a long-term follow-up. However, in the present study, we have been able to assess the main objective of our research, which was to compare the efficiency of HPV integration test and HPV testing to predict recurrence after CIN2-3 treatment.

HPV-integration-positive predicts higher rates of recurrence in CIN2-3 women, and may serve as a prognostic factor. Furthermore, PHISL between pre-treatment and post-treatment is a powerful predictor for postoperative residual/recurrent disease in HPV-integration-positive CIN2-3 patients, and is more accurate than HPV testing and cone margin status. This could reduce unnecessary colposcopies and cervical biopsies, which bring about considerable psychological stress and physical discomfort. Furthermore, accurate prediction with PHISL is of great importance in areas where colposcopy resources are scarce. More studies are expected to confirm the data of this new method and to evaluate the cost-effectiveness of HPV integration tests in the follow-up of patients treated for CIN2-3.

### Supplementary Information

Supplementary Material 1.

## Data Availability

The data that support the findings of this study is available from the first author (Fanwei Huang) upon request.
