# Supplementary material for: HPV integration: a precise biomarker for detection of residual/recurrent disease after treatment of CIN2-3
Source: Infect Agent Cancer. 2024 Aug 8;19:36. doi: 10.1186/s13027-024-00600-8 (PMC11308599; doi:10.1186/s13027-024-00600-8)
Supplement: Supplementary file 1 — Supplementary Material 1. [file 13027_2024_600_MOESM1_ESM.docx]

**Table S1. Integration characteristics of Persistent HPV integration at the same breakpoint**

| No. of patients | HPV genotype | Prognosis | Breakpoints in Human chromosomes | | | | Follow-up | |
| --- | --- | --- | --- | --- | --- | --- | --- | --- |
|  |  |  | Chromosome | Breakpoint | Gene | Gene name | Colposcopy examination results | Time between the preoperative and postoperative integration tests （months） |
| 1 | HPV51 | Recurrence | 17 | 46767068 | Intronic | WNT3 | CIN3 | 6 |
| 2 | HPV16 | No recurrence | 10 | 118794427 | Intergenic | CACUL1, NANOS1 | Normal | 12 |
| 3 | HPV16 | Recurrence | 2 | 60462776 | Intronic | BCL11A | CIN3 | 4 |
| 4 | HPV16 | Recurrence | 11 | 66965015 | Intergenic | PC, C11orf86 | CIN3 | 6 |
| 5 | HPV33，HPV52 | Recurrence | 12 | 107114740 | Intergenic | CRY1, BTBD11 | CIN3 | 9 |
| 6 | HPV16 | Recurrence | 15 | 58436008 | NcRNA_exonic | LIPC-AS1 | CIN3 | 18 |
| 7 | HPV16 | Recurrence | 16 | 866867 | Intronic | LMF1 | CIN2 | 5 |

HPV, human papillomavirus; CIN, cervical intraepithelial neoplasia.
